# Item

**Published:** 1902-12

**Authors:** 


### ITEM.

Monument for Rudolf Virchow.—The German committee in charge of the celebration in honor of Rudolf Virchow's eightieth birthday—Prof. Waldeyer, chairman; Prof. Posner, secretary—has begun collecting funds for the purpose of erecting a monument in memory of that great and unique man and physician. The undersigned are anxious and ready to receive contributions which will be duly acknowledged. Frank Billings, president of the American Medical Association, 100 State St., Chicago; Thomas D. Coleman, 505 Greene St., Augusta; A. Jacobi, 19 East 47th St. New York City; W. W. Keen, president of the congress of American Physicians and Surgeons, 1729 Chestnut St., Philadelphia; Wm. H. Welch, 935 St. Paul St., Baltimore.

